# Ventricle-specific epicardial pressures as a means to optimize direct cardiac compression for circulatory support: A pilot study

**DOI:** 10.1371/journal.pone.0219162

**Published:** 2019-07-05

**Authors:** Jooli Han, Matthew Kubala, Edgar Aranda-Michel, Dennis R. Trumble

**Affiliations:** Department of Biomedical Engineering, Carnegie Mellon University, Pittsburgh, Pennsylvania, United States of America; University of Alabama at Birmingham, UNITED STATES

## Abstract

Direct cardiac compression (DCC) holds enormous potential as a safe and effective means to treat heart failure patients who require long-term, or even permanent, biventricular support. However, devices developed to date are not tuned to meet the individual compression requirements of the left and right ventricles, which can differ substantially. In this paper, a systematic study examining the relationship, range, and effect of independent pressures on the left and right epicardial surfaces of a passive human heart model was performed as a means to optimize cardiac output via DCC support. Hemodynamic and tissue deformation effects produced by varying epicardial compressions were examined using finite element analysis. Results indicate that 1) designing a direct cardiac compression pump that applies separate pressures to the left and right ventricles is critical to maintain equivalent stroke volume for both ventricles, and 2) left and right ventricular epicardial pressures of 340 mmHg and 44 mmHg, respectively, are required to induce normal ejection fractions in a passive heart. This pilot study provides fundamental insights and guidance towards the design of improved direct cardiac compression devices for long-term circulatory support.

## Introduction

Heart failure (HF) is a highly debilitating disease that accounts for 17.9 million deaths each year worldwide.[1] The etiology of this disease ranges widely from coronary artery disease, valve disease and high blood pressure (hypertension) to myocarditis, arrhythmias, diabetes and obesity. The symptoms also vary extensively from nausea, shortness of breath, and fatigue to pulmonary edema and organ failure, but all result from insufficient cardiac output that can, in principle, be restored to normal levels with the use of an implantable blood pump.[2] Unfortunately, despite the fact that a wide variety of such devices are currently available to assist the failing heart, the five-year survival rate for these patients remains barely above 50% after initial diagnosis.[3] The reasons behind this include bleeding, driveline infection, and inadequate aftercare. Furthermore, pump thrombosis, where blood clots form at blood-device interfaces, remains among the most common (and most dangerous) device related complications due to the fact that arterial thromboemboli can damage virtually any tissue in the body, including the brain (stroke), lungs (pulmonary embolism), and kidneys.[4]

To boost cardiac output without risking thrombotic events associated with blood-contacting surfaces, there have been numerous developments of non-blood-contacting ventricular assist devices (VADs) that apply pressure to the exterior surface of the heart such as the Anstadt Assistor Cup, DeBakey’s pneumatic compression cup, CorInnova’s minimally invasive direct cardiac compression (DCC) sleeve and a biomimetic silicone sleeve that both compresses and twists the heart.[5–7] Surprisingly, despite the large body of work centered around biventricular compression and the obvious differences in left/right ventricular anatomy and systemic/pulmonary afterload pressures, there has never been a systematic study examining the application of independent pressures over the left and right ventricles as a means to optimize cardiac output in heart failure patients. In this study, our goal is to quantify pressure requirements by the DCC sleeve for clinical level improvements in both ventricles for effective long-term circulatory support. As a first step toward that goal, we examined the hemodynamic and tissue deformation effects produced by varying epicardial pressures (EPs) on the left and right ventricles in a passive finite element model of the human heart.

## Materials and methods

### Biventricular model geometry

**Fig 1:**
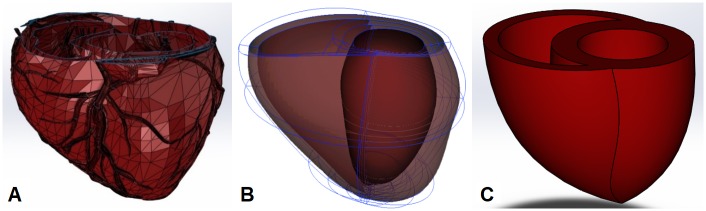
Human biventricular models. Examples of human biventricular models reconstructed from a CT scan **(A)**; an echocardiogram scan **(B)**; and a computer rendering of a passive human biventricular model **(C)** designed with these reported data.

Using data from computerized tomography (CT) stereolithography scans (Fig 1A) of human hearts downloaded from open-source 3D CAD model depositories such as GrabCAD (www.grabcad.com) and 3D CAD Browser (www.3dcadbrowser.com), a 3D echocardiogram model (Fig 1B) that was scanned at different directions and reconstructed by Insilicomed Inc. (La Jolla, CA), and eight different literatures that reported average dimensions of human heart, a biventricular (BiV) model (11.6 cm (*w*) x 13.2 cm (*l*) x 10.5 cm (*h*)) (Fig 1C) with a prolate left ventricle (LV) and a crescent-shaped right ventricle (RV) with wall thicknesses of 1.6 cm and 0.9 cm, respectively, was rendered *in silico* using SolidWorks 3D design software (Dassault Systèmes SolidWorks Corporation, Waltham, MA).[8–15] The initial ventricular volumes prior to cardiac compression were set to normal end-diastolic values of 135 mL for the LV and 150 mL for the RV so that a stroke volume (SV) of 80 mL corresponds to roughly 60% left ventricular ejection (LVEF) and 53% right ventricular ejection fraction (RVEF).[11] This computer-rendered BiV model was then converted to an initial graphics exchange specification (.IGES) format for cardiac compression finite element analysis (FEA) simulations.

### Constitutive modeling of passive heart tissue

A multiscale FEA software platform called Continuity, developed by the Cardiac Mechanics Research Group at University of California San Diego (UCSD), was used to estimate the passive constitutive law of the myocardium employed in these simulations. Continuity and the derivative work, Continuity Pro (Insilicomed Inc.), combine high-order FEA of a nonlinear constitutive law for the muscle fibers and a dynamic model of myocardial excitation-contraction coupling. The software was used to inflate a passive cylindrical model of cardiac tissue, which was extracted from a cohort of 13 patient-specific computational models based on medical imaging and other clinical measurements obtained from patients.[16] A transversely isotropic constitutive law, adapted from previous work done by Guccione et al., was employed without any calcium activation.[17] The original constitutive law was developed by using large deformation theory and previous stress-strain experiments on canine hearts to estimate parameters. The law employed here is a Fung type hyperelastic model with an exponential strain energy density function (Eq 1).

$$W=\frac{C}{2}(e^{Q}-1)$$ (1)

in which *C* is a stress scaling coefficient, and *Q* is a quadratic function of the six normal strains and associated shear strains of a symmetric, Lagrangian finite strain tensor. Q has the following form:

$$Q=b_{2}{E_{ff}}^{2}+b_{3}({E_{cc}}^{2}+{E_{rr}}^{2}+2*E_{cr}*E_{rc})+b_{4}({2*E}_{rf}*E_{fr}+2*E_{fc}*E_{cf})$$ (2)

The coordinate system for the strain tensor is the orthonormal basis of the local fiber coordinate system, with each fiber having a direction along its axis (*f*), a direction perpendicular to the fiber and directed along the surface of the heart (*c*), and a direction also perpendicular to the fiber directed transmurally (*r*). *E*_*ff*_, *E*_*cc*_, and *E*_*rr*_ are the normal strains in the fiber, cross-fiber, and transmural or radial directions respectively. Likewise, *E*_*rc*_, *E*_*rf*_, and *E*_*fc*_ are the shear strains. The variables *b*_1_ to *b*_4_ are absolute constants that scale the various strains. All the constants are outlined in previous studies conducted with Continuity Pro but a few are reiterated here for convenience (Table 1).[16]

**Table 1:** Parameters used in the passive constitute law of myocardial fibers.

Parameter	Value
Stress Scaling Coefficient (kPa) [C]	0.80
Fiber Strain Coefficient [*b*_2_]	18.50
Transverse strain coefficient [*b*_3_]	3.58
Fiber-transverse shear coefficient [*b*_4_]	1.63

**Fig 2:**
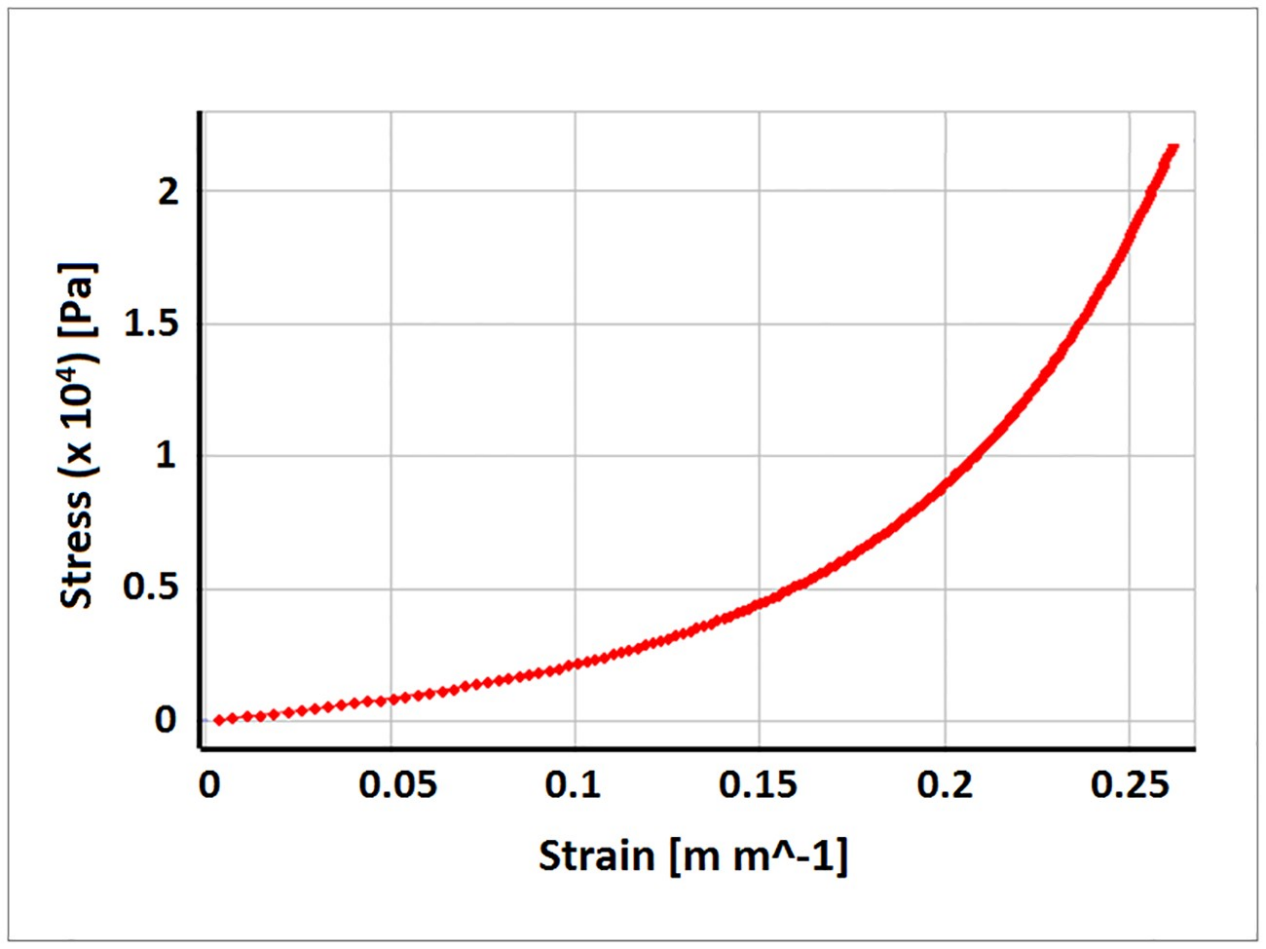
Stress-strain curve of the heart tissue data overlay with Yeoh 3^rd^ model.

In the cylinder, the fiber direction rotates helically from -37 degrees on the epicardium to +83 degrees on the endocardium with respect to the circumferential direction. The inner wall of the cylinder was subjected to a linear increase in internal pressure up to 2 kPa. The strain of the middle layer of the myocardium was calculated to estimate the effect of the rotating fiber directions. This strategy was selected as it would be almost impossible to recapitulate the fiber directions in ANSYS and an aggregate estimation would be sufficient for a first order approximation. This stress-strain relation (Fig 2) was imported into ANSYS Mechanical and fit with a 3^rd^ order Yeoh hyperelastic equation for the strain energy density as depicted in (Eq 3). The Yeoh form was chosen as it deals well with incompressible nonlinear elastic materials, its dependence on only the first invariant of the Cauchy-Green deformation tensor, and the ability to fit the relation well with polynomial shaped stress-strain curves.

$$W=\sum_{i=1}^{3}C_{i}{\left(I_{1}-3\right)}^{i}$$ (3)

Again, W is the resulting strain energy, *C*_*i*_ are constants determined empirically and *I*_1_ is the first invariant of the Cauchy-Green deformation tensor. *I*_1_ reduces to $\lambda_{1}^{2}+\lambda_{2}^{2}+\lambda_{3}^{2}$, in which lambda_1_, lambda_2_ and lambda_3_ are the three principal stretches in which the principal axes coincide with the global coordinates of the cylinder, i.e., the circumferential, axial and radial coordinate directions. The expanded form of the Eq 3 is presented below (Eq 4).

$$W=C_{1}(I_{1}-3)+C_{2}{\left(I_{1}-3\right)}^{2}+C_{3}{\left(I_{1}-3\right)}^{3}$$ (4)

To estimate the parameters, the stress-strain curve was fit to the strain energy density function assuming the applied pressure was causing an equibiaxial extension of the tissue. The resulting parameters from this fit are: *C*_*1*_ = 2,734 Pa, *C*_*2*_ = 15,113 Pa and *C*_*3*_ = 89,498 Pa. The goodness of fit is illustrated in Fig 2 and shows good agreement with the calculated stress-strain curve. This constitutive heart tissue model was then evenly applied to the BiV model (Fig 1C) to imitate human heart tissue material properties without any myocardium layers or tissue fiber directions.

### Boundary and loading conditions

**Fig 3:**
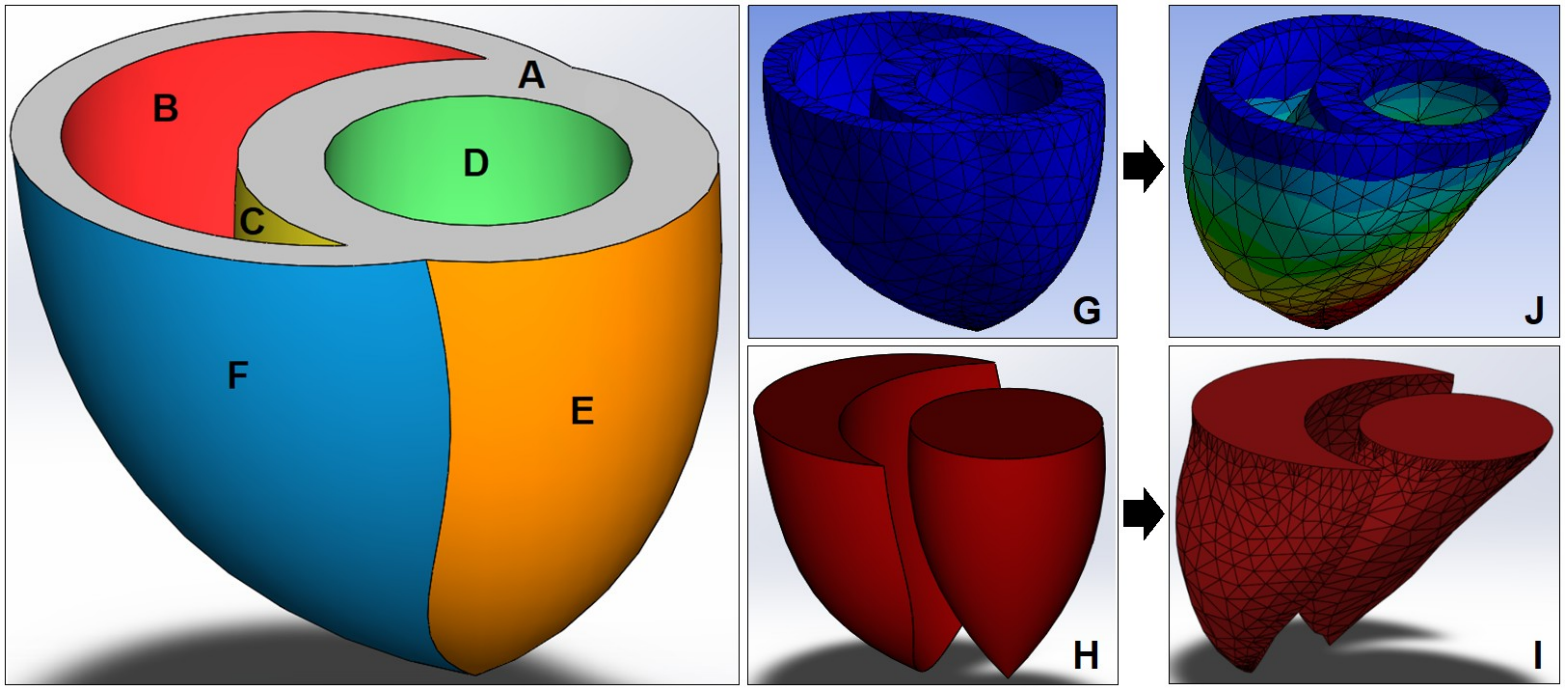
Computer renderings of a biventricular model. The BiV model surface divided into six sections for proper boundary and loading conditions applications (**A**: Fixed top surface, **B, C**: RVP, **D**: LVP, **E**: LVEP, **F**: RVEP), and generated into a mesh model **(G)** for cavity extractions before **(H)** and after **(I)** deformations **(J)**.

Boundary and loading conditions were applied to the BiV model prior to running static structural FEA using ANSYS Workbench. The top surface of the model (Fig 3A) was set as a fixed support to mimic the natural constricted movement of the valvular plane of the heart. A small contact offset of 0.001m was added to the interior of the RV to prevent the inner surfaces (Fig 3B and 3C) from completely collapsing or penetrating each other. Afterload pressures were applied to the interior surfaces of the LV (Fig 3D) and RV (Fig 3B and 3C), and epicardial pressures (EPs) were applied to the exterior surfaces of the LV (Fig 3E) and RV (Fig 3F).

In this study, we investigated the range and relationship between applied external pressures and BiV model deformation under four different case scenarios: 1) uniform EP at zero afterload, 2) separate LVEP and RVEP at zero afterload, 3) uniform EP at end-systolic afterloads, and 4) separate LVEP and RVEP at end-systolic afterloads. For Cases 1 and 3, a uniform EP was evenly applied to the entire epicardial surface (Fig 3E and 3F) to simulate current direct cardiac compression methods. For Cases 2 and 4, LVEP and RVEP were applied to the epicardium of the LV (Fig 3E) and RV (Fig 3F) separately to simulate discrete LV and RV compressions. For zero afterload cases, both LV (Fig 3D) and RV (Fig 3B and 3C) afterloads were set to 0 mmHg to test the ventricular behavior solely dependent on its tissue material properties. For end-systolic cases, LV (Fig 3D) afterload was set to an aortic pressure (AoP) of 100 mmHg and RV (Fig 3B and 3C) afterload was set to a pulmonary artery pressure (PAP) of 25 mmHg to simulate direct cardiac compressions of a completely passive heart under normal circulatory pressure conditions.[18–20]

### Mesh cavity extraction for SV and EF analyses

To assess the effects of various loading conditions on the cardiac output of the BiV model, both initial and deformed mesh models (Fig 3G and 3J) were exported as stereolithography (.STL) files and intersected for cavity extractions (Fig 3H and 3I) using SolidWorks. The stroke volumes were computed by subtracting the extracted final LV and RV cavity volumes from the end-diastolic LV and RV volumes before compression (Eq 5). Ejection fractions were calculated by dividing the computed SVs by the initial end-diastolic volumes (Eq 6).

StrokeVolume(SV)=EndDiastolicVolume−FinalCavityVolume (5)

$$Ejection\;Fraction\;(EF)=\frac{End\;Diastolic\;Volume-Final\;Cavity\;Volume}{End\;Diastolic\;Volume}\;x\;100$$ (6)

## Results

### Geometric deformation and cardiac output

**Fig 4:**
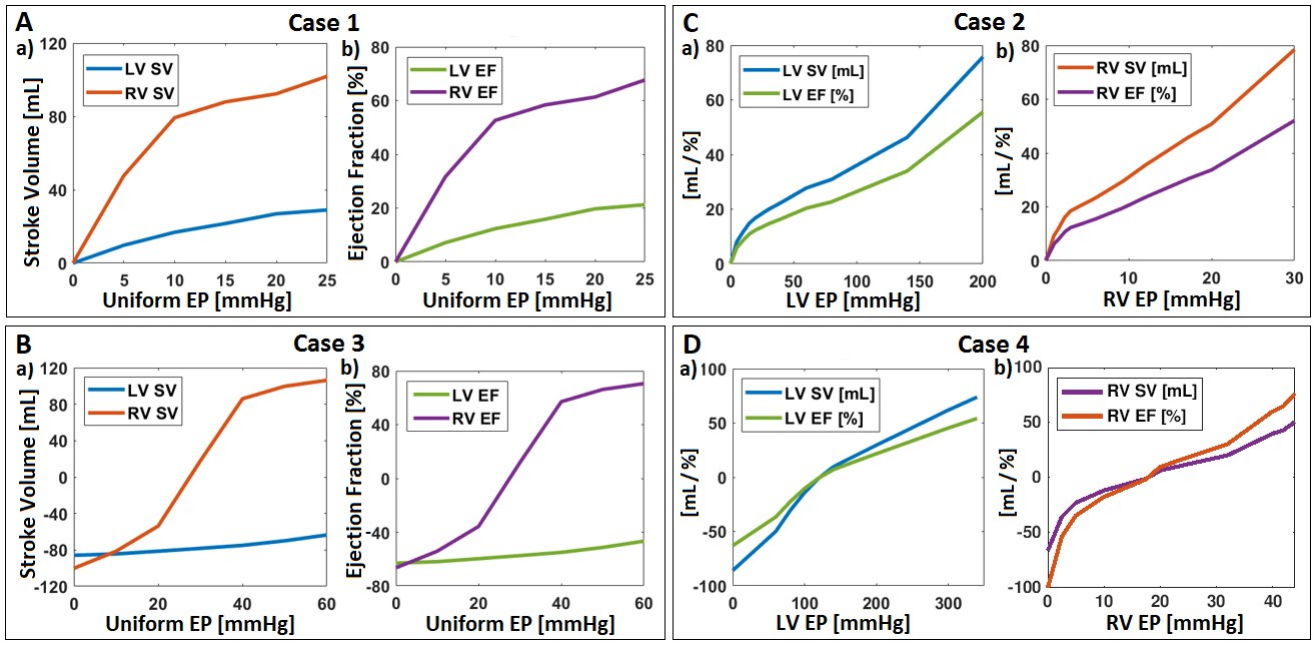
Stroke volumes and ejection fractions of the BiV model after four-case cardiac compressions. **A**: Stroke volumes **(a)** and ejection fractions **(b)** of Case 1 where uniform EPs were applied to both ventricles with zero afterload; **B**: Stroke volumes **(a)** and ejection fractions **(b)** of Case 3 where uniform EPs were applied to both ventricles with end-systolic afterloads; **C**: Cardiac outputs of Case 2 where separate EPs were applied to LV **(a)** and RV **(b)** with zero afterload; and **D**: Cardiac outputs of Case 4 where separate EPs were applied to LV **(a)** and RV **(b)** with end-systolic afterloads.

**Fig 5:**
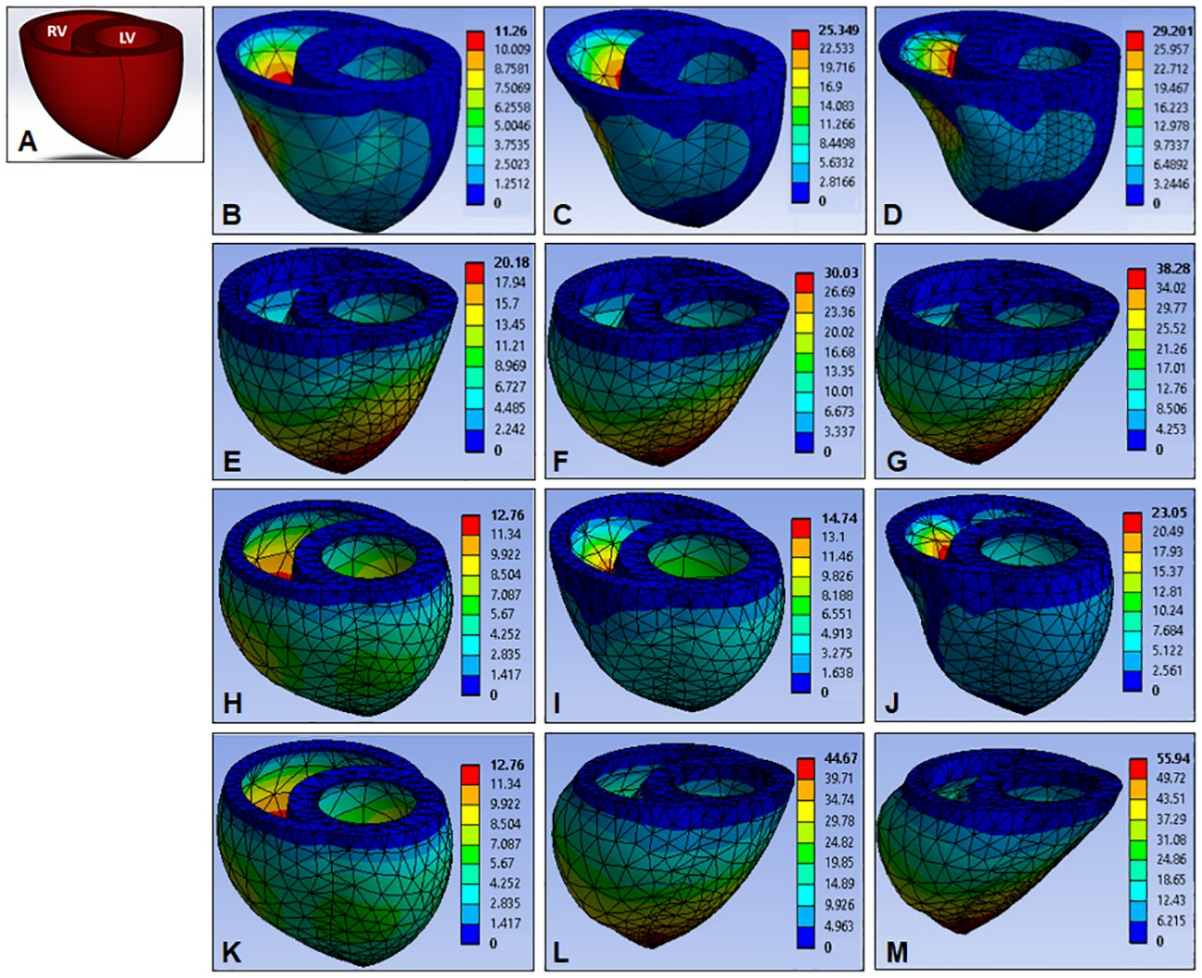
The BiV model (A) Deformations after four-case cardiac compressions. **Case 1**: A uniform EP of 5 mmHg **(B)**, 10 mmHg **(C)**, and 25 mmHg **(D)** applied to both ventricles with zero afterload; **Case 2**: Different combinations separate EPs (5 mmHg LVEP and 2 mmHg RVEP **(E)**, 30 mmHg LVEP and 4 mmHg RVEP **(F)**, 60 mmHg LVEP and 10 mmHg RVEP **(G)**) applied to both ventricles with zero afterload; **Case 3**: A uniform EP of 0 mmHg **(H)**, 30 mmHg **(I)**, and 60 mmHg **(J)** applied to LV and RV with end-systolic afterloads; and **Case 4**: Different combinations separate EPs (0 mmHg LVEP and 0 mmHg RVEP **(K)**, 100 mmHg LVEP and 10 mmHg RVEP **(L)**, 160 mmHg LVEP and 25 mmHg RVEP **(M)**) applied to LV and RV with end-systolic afterloads. Deformation units are in mm.

For Cases 1 and 3 (Fig 4A and 4B), we reported LV and RV deformations after uniform EPs were applied to the epicardial surfaces. For Cases 2 and 4 (Fig 4C and 4D), we investigated left and right EP combinations that induced comparable left and right SVs and EFs until the EF reached 50 ~ 55%, which is considered a normal value for both LV and RV.[8,16] The BiV model deformations of all four cases are illustrated in Fig 5.

### Uniform EP at zero afterload

For Case 1, a range of EPs were evenly applied to both ventricles with no afterloads inside to simulate a scenario where a symmetrical cardiac compression device pumps the ventricles of a completely passive, isolated heart. When the EPs ranging from 0 to 25 mmHg were applied uniformly across the epicardial surface, LVSV increased from 0 mL to 28.99 mL while RVSV increased from 0 mL to 101.93 mL (Fig 4A-a), inducing up to 21.27% LVEF and 67.68% RVEF (Fig 4A-b). During compressions, the maximum deformation occurred at the midsection of the RV wall (Fig 5B–5D), resulting in significantly more blood displacement from the RV than the LV (mean RVEF/LVEF ratio = 3.06).

### Separate EPs at zero afterload

**Fig 6:**
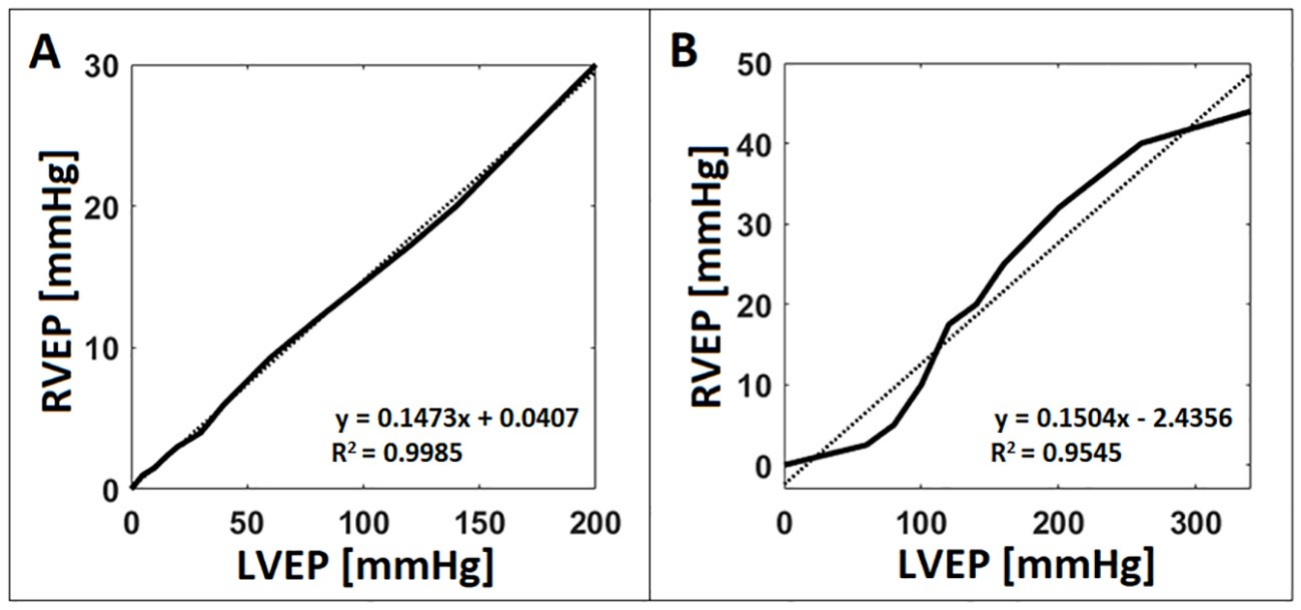
The RVEP-to-LVEP ratio for zero and end-systolic afterloads. The relationship between applied LVEPs and RVEPs to induce the similar amounts of LVEFs and LVEFs (Δ <5%) for ventricles with zero **(A)** and end-systolic **(B)** afterloads.

In Case 2, two individual EPs were applied to the LV and RV with no pressure afterload inside the heart. We looked for pressure combinations that induced similar LV and RV outputs (difference less than 5%) until EFs exceeded 50%. The LVEP ranging from 0 to 200 mmHg induced LVSVs and LVEFs up to 75.77 mL and 55.59% respectively (Fig 4C-a), while RVEPs ranging from 0 to 30 mmHg induced the RVSVs and RVEFs up to 78.48 mL and 52.11% respectively (Fig 4C-b). The maximum deformation during compression occurred at the apex of the heart (Fig 5E–5G), which was due to shifts of the septal wall and the BiV model towards the RV in reaction to the much higher LVEP compared to the RVEP while maintaining similar LV and RV outputs. The relationship between LVEP and RVEP was close to linear with a mean RVEP/LVEP ratio of 0.1473 (Fig 6A). These data indicate that it takes roughly 6.79 times the pressure to compress the LV than the RV for a completely passive heart model with zero afterload.

### Uniform EP at end-systolic afterloads

Although no cardiac compression device will normally have to support a completely passive heart since even the most severe heart failure cases exhibit 15 ~ 30% EFs, we tried compressing the passive BiV model against end-systolic afterloads (i.e., AoP = 100 mmHg, PAP = 25 mmHg) as an indication of what might occur in a ‘worst-case’ scenario where ventricular fibrillation occurs during DCC support. Again, for Case 3, applications of uniform EPs to both ventricles represented cardiac compressions of a radially symmetrical pump. When EPs ranging from 0 to 60 mmHg were applied evenly to the epicardium, LVSV increased from -85.90 mL to -63.60 mL and RVSV increased from -100.20 mL to 106.30 mL (Fig 4B-a). This corresponded to LVEF inflation from -63.02% to -46.66% and RVEF inflation from -66.53% to 70.58% (Fig 4B-b). Both ventricles started out in a bulged-out form with negative EFs because the afterload was initially higher than the compression pressures outside. The LVEF remained in the negative range while RVEF passed +70% with 60 mmHg EP. The inward deformation of the RV wall was significantly more drastic compared to the LV wall (Fig 5H–5J), which caused a dramatic difference in LV and RV cardiac outputs (mean RVEF/LVEF ratio = 9.34).

### Separate EPs at end-systolic afterloads

When LVEP and RVEP were applied separately to look for EP combinations that induce comparable LV and RV outputs (difference less than 5%), LVEPs ranging from 0 to 340 mmHg induced LVSVs and LVEFs up to 73.86 mL and 54.19% respectively (Fig 4D-a), while RVEPs ranging from 0 to 44 mmHg induced RVSVs and RVEFs up to 76.40 mL and 50.73% respectively (Fig 4D-b). Again, the maximum deformation occurred at the apex of the heart (Fig 5K–5M) due to the BiV model shift towards the RV to compensate for higher pressures over the LV. In this case the RVEP-to-LVEP ratio was roughly 0.1504 (Fig 6B), which means it requires about 6.64 times higher pressures on the LV than the RV to maintain similar LV and RV outputs.

### Stress, strain, and energy

**Fig 7:**
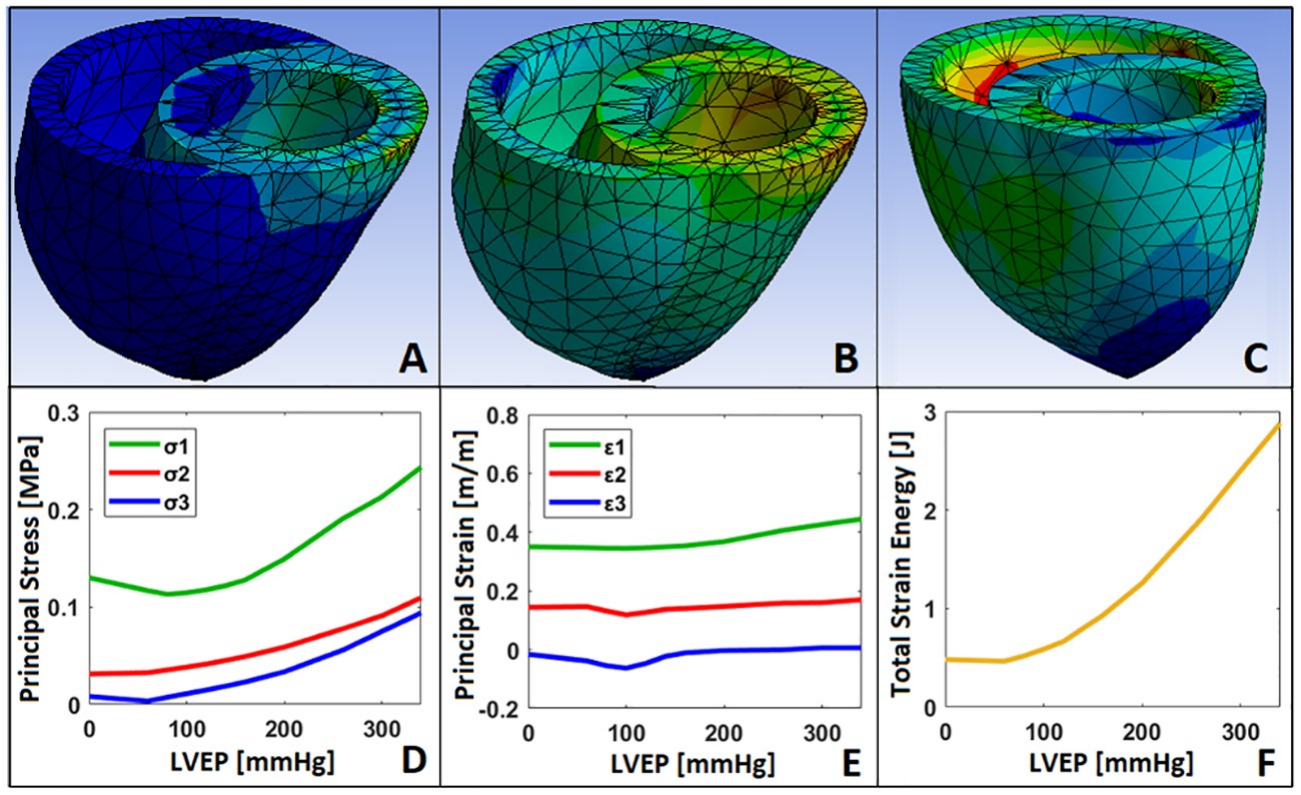
Stress, strain, and energy experienced by the BiV model for Case 4. Local maximum (σ_1_), middle (σ_2_) and minimum (σ_3_) principal stresses and maximum (ε_1_), middle (ε_2_) and minimum (ε_3_) principal strains and total strain energy were solved using ANSYS Workbench for Case 4 where separate LVEP and RVEP were applied to the LV and RV loaded with end-systolic AoP and PAP, respectively. Principal stresses **(A)** initially dropped before rising due to the higher ventricular afterload compared to epicardial compressive pressure **(D)**. Similarly, principal strains **(B)** dropped until the EP exceeded afterload pressures **(E)**. Strain locale transitioned from the fixed top surface **(B)** to the middle of the RV wall **(C)** as EP increased. Total strain energy of the BiV model exhibited an exponential trend due to the hyperelastic material property of the model **(F)**.

Local maximum principal stresses and strains and total strain energy experienced by the BiV model were studied for Case 4 since it is the simulation closest to physiologic cardiac compression. When maximum (σ_1_), middle (σ_2_) and minimum (σ_3_) principal stresses and maximum (ε_1_), middle (ε_2_) and minimum (ε_3_) principal strains were computed using ANSYS, both max stress and strain were initially located near the top surface of the BiV model due to the fixed boundary condition restricting movement (Fig 7A and 7B). As the BiV model was compressed with higher EPs, however, the maximum strain locale transitioned to the middle of the RV wall (Fig 7C). Also, because the BiV model is in a bulged-out state for LVEPs between 0 and 100 mmHg, the maximum principal stress initially dropped until the EPs exceeded the ventricular afterloads (i.e., LVP = 100 mmHg and RVP = 25 mmHg) (Fig 7D). Similarly, all three principal strains (ε_1_, ε_2_ and ε_3_) dropped before rising starting from 100 mmHg LVEP (Fig 7E).

The total strain energy stored in the BiV model due to deformation was also computed by summing up probed energies for each mesh element using ANSYS Workbench (Fig 7F). The total strain energy experienced by the model was 0.483 J at 0 mmHg EPs and reached up to 2.88 J with 340 mmHg LVEP and 44 mmHg RVEP compressions. The strain energy is never zero because the BiV model experiences outward bulging even without any external compression (Fig 5K), and it increases with an exponential trend due to Yeoh’s hyperelastic material properties of the model. The work in the LV and RV blood displacements at 54.2% LVEF and 50.7% RVEF were 0.985 J and 0.305 J, respectively, when hand-calculated by multiplying the afterloads with the corresponding volume displacements (Eqs 7 and 8).

$$W_{LV\_blood\;}=End\;Systolic\;AoP\;x\;\Delta V_{LV\_blood}$$ (7)

$$W_{RV\_blood\;}=End\;Systolic\;PAP\;x\;\Delta V_{RV\_blood}$$ (8)

These results indicate that 2.88 J are required for elastic deformation of the BiV model on top of 0.985 J and 0.305 J for LV and RV blood displacements in order to induce normal EFs. But because this simulation was modeled around several unavoidable assumptions, it does not necessarily mean the direct cardiac compression device needs to exert 4.17 J of energy for proper therapeutics (see Limitations section below).

## Discussion

### Independent LV and RV epicardial pressures

Despite a number of investigations differentiating LV and RV epicardial compressions, including an inflatable “Heart Patch” device consisting of two separate patches placed on the left and right ventricular free walls of a sheep heart and a soft robotic cardiac compression sleeve that selectively actuates LV and RV, no previous research studies have systematically examined the relationship, range, and effect of individual LV and RV epicardial compressions on a human cardiac model.[16,21]

As the gap between the LV and RV epicardial compression requirements was found to be not only present but also rather large (Table 2) due to the thinner wall thickness and lower afterload of the RV than those of the LV, it is safe to argue that separate applications of EPs on the LV and RV are critical to maintaining comparable SVs in both ventricles.[13,14] However, because no patient heart will be completely passive prior to the implantation of a DCC support device, the natural myocardial contraction and thickening dynamics of the beating heart will certainly reduce the amount of EPs required in practice. Therefore, the LVEP of 340 mmHg and RVEP of 44 mmHg requirements reported in this study represent an upper bound on the epicardial pressures needed to support a failing heart.

**Table 2:** A summary table of LVEP, RVEP and total strain energy requirements for LV and RV ejection fractions higher than 50% for both Case 2 and Case 4.

	Case 2		Case 4	
**LV**	LVEP	200 mmHg	LVEP	340 mmHg
	LVEF	55.59%	LVEF	54.19%
**RV**	RVEP	30 mmHg	RVEP	44 mmHg
	RVEF	52.11%	RVEF	50.73%
**Total E**	2.15 J		4.17 J	

The difference between energy requirements for compressing against zero (Case 2) and systolic (Case 4) afterloads was another interesting finding of this study. Because epicardial compression had to work against both elastic deformations of the BiV model and afterload pressures inside the ventricles, Case 4 required about twice as much energy as Case 2 (Table 2).

### Limitations of the BiV model simulations

Although results from these preliminary simulations suggest that a cardiac compression device must deliver 2.88 J to deform the myocardium and an additional 1.29 J to move the blood against typical arterial afterloads in order to achieve a normal level of cardiac output in a passive heart (Case 4), it is important to point out that numerous assumptions and simplifications were made for this simulation study. Because these analyses were done on a simplified model with several underlying assumptions, these data do not necessarily indicate that an effective cardiac compression device must be designed to match these energy requirements.

The reasons are several. First of all, the geometry of the BiV model was designed based upon eight individual reference studies that report measurements of human heart dimensions. Due to substantial variations in patient size and measurement techniques, the BiV model used here is an idealized amalgamation and, like any such model, cannot profess to represent all patients of all stages of heart failure. Secondly, these simulations were done on a completely passive heart model that produces zero EF on its own, which, of necessity, will never be the case in clinical practice. According to New York Heart Association (NYHA) Classifications, Class IV heart failure patients (the most severe category) have an LVEF under 30%.[22] Considering some level of active myocardial contraction is present even for Class IV end-stage heart failure patients, the energy requirement computed with a perfectly inactive model could be an overestimation. At the same time, due to the natural cardiac muscle thickening and tissue stiffening during systole, the energy requirement results may be an underestimation. Although this study was conducted with considerable simplifications, there is no doubt that this pilot study is an incremental, and yet, crucial addition towards the development of an effective DCC device for long-term circulatory support. A more advanced simulation model that takes essential physiological phenomena—like myocardial stiffening and thickening during systole, competence of the atrioventricular and semilunar valves, variations in ventricular geometry, septal wall movements and hypertensive afterloads—into account will be a pivotal next step.

### Cardiac compression device design and future steps

The DCC sleeve under development is a patient-specific device. Each device will be customized via fitting around a 3D reconstruction of a scanned patient heart. Repeating the cardiac compression simulations with a high-order biventricular model of a beating heart via multi-scale FEA using Continuity Pro (Insilicomed, Inc.) and hemodynamic studies using fluid structure interaction analysis will provide a more accurate understanding of compression requirements and cardiac behaviors as well as ensure the viability of the device for various extreme cases such as hypertrophic cardiomyopathy.[16] Once these requirements are further determined, a direct cardiac compression device that meets those criteria can be designed. One potential incarnation currently under development in our lab, a muscle-powered soft-robotic cardiac compression sleeve, is a completely tether-free ventricular assist system that compresses the epicardial surface of the heart and is powered by endogenous skeletal muscle. This device captures the contractile work of latissimus dorsi muscle and converts it into hydraulic power to actuate a pulsatile soft robotic pump, thereby enabling fully implantable circulatory support without the risk of driveline infections or thromboembolic events.[23]

As a preliminary exploration, this study provides fundamental insights and guidance towards the design of improved DCC devices for long-term circulatory support. The observations regarding resistance produced by both myocardial walls and pulmonary and arterial afterload pressures, and the relationship between LV and RV compression energy requirements, suggest that designing a two-part compression system that supports the LV and RV independently from each other will most effectively improve cardiac function.

## Supporting information

S1 File An Excel sheet that shows all relevant data to support our findings for Cases 1, 2, 3, and 4. (XLSX)
